# Supplementary material for: Revision of the Assassin Bug Genus Sigicoris stat. nov. Based on Morphological Study and Molecular Phylogeny (Heteroptera: Reduviidae: Peiratinae)
Source: Insects. 2022 Oct 19;13(10):951. doi: 10.3390/insects13100951 (PMC9604541; doi:10.3390/insects13100951)
Supplement: Supplementary file 1 [file insects-13-00951-s001.zip › Table S1.pdf]

Table S1. Information of newly-sequenced species in the present study.

Species	Label information of voucher specimen	Original depository
Androclus borneensis	Malaysia, Sabah, Borneo Jungle Girl Camp, 2017-II-12, Liu Yingqi	CAU
Androclus granulatus	Coll. I.R.Sc.N.B. Enggano lsl., cca 120km W or Bengkulu, v. 2005, 100m, local collectors I.G. 31. 127	IRSNB
Brachysandalus lurco	25.X.2017 Australia Queensland-Brisbane Sunshine Coast-Cooroy Miloslav Mylek Lgt	CAU
Calistocoris caesareus	Malaysia, Sabah, Borneo Jungle Girl Camp, 2016-IV-30, Light, Leg. Li Hu	CAU
Catamiarus brevipennis	12/09 Dharwar. H. Swale., Catamiarus brevipennis Serv. M.L. Cook det. 1974	NHM
Ectomocoris luridus	SOUTH AFRICA: NATAL UKULU RIVER 2843S 3154E, 11.86 P ATKINSON, AT LIGHT	NHM
Ectomocoris quadriguttatus	Loc. Palayankottai	CAU
Ectomocoris quadrimaculatus	Republic of Uganda Elizabeth 2018-II-25 LI Hu	CAU
Ectomocoris xanthopus	N. NIGERIA: Zaria, Samaru. 20-ix-196, J. C. Deeming m. v. trap., Ectomocoris xanthopus Schaum det., Brit. Mus. 1967-684	NHM
Eidmannia guyanensis	British Guiana Cattle Tail Suivey Takaruni R. (sec. 2) June 1919. a.a. ahaham. Coll, Brit. Mus. 1965-275, NHMUK 013586408	NHM
Fusius dilutus	NIGERIA: lbadan, Moor Plantation. 22. iii. 1956. V. F. Eastop. B.M. 1956-283.	NHM
Fusius rubricosus	SOUTH AFRICA: Transvaal n. Kruger National Park Pafuri. 22°27'S 31°17'E 21/1 1979 L. Braack ex. Impala carcass	NHM
Sigicoris brumalis comb. nov.	Stn. No. 46., NEW GUINEA: Madang Dist., Finisterre Mts., Damanti 3,550 ft. 2-11. x. 1964	NHM
Sigicoris dominiqueae sp. nov.	Coll. I. R. Sc. N. B. Canopy Mission Papua Neu Guinea (Madang prov): Baiteta 04. VI. 1996 Light trap AR7 Leg. Olivier Missa	IRSNB
Sigicoris sexguttatus comb. nov.	Indonesia, West Papua ARFAK MTS, 1190 m alt DUEBEI ENV, 21. 1-8.2.2008 cca 20 km S of Warmere	CAU

	Manokwari distr, St Jakl lgt	
Lamotteus ornatus	COLL. MUS. TERVUREN Côve d'ivoire : Bingerville V. 1962 J. Decelle, Lamotteus ornatus Vill. A. Villiers det 1965	RMCA
Lestomerus affinis	2010-VII-22 INDIA Andhra Pradesh Neuore District Naidupet mandal Dwarakapuram village C-C-Chen leg.	CAU
Lestomerus sanctus	INDIA Andhra Praclesh Nedore District Naidupet mandal Dwarakapuram village c-c chen 2013	CAU
Melanolestes picipes	2012-IV-28 Robinson Lexington KY, USA leg. Hu Li	CAU
Microsandalus umbrosus	H. Hacker. Bribie Isd Dec 1920, Queensland. H. Hacker. B.M. 1924-455., 94, NHMUK 013588785	NHM
Neopirates nyassae	A. Turner Sekoke. July. 1932, NHMUK 013588798	NHM
Oblongiala zimbabwensis	Oblongiala zimbabwensis Det. Yingqi Liu, ZIMBABWE: A. Watsham B.M. 1985-248, Oct. 1984, NHMUK 013588801	NHM
Pachysandalus collaris	Env. De Fort-Lamy Farcha 6.IV, Rép. du. Tchad Bas-Chari, 1964 J. Gruvel, coll.	MNHN
Parapirates cachani	TAFO GHANA 7:III:66 LESTON, UV TRAP, 862, Parapirates cachani Vill. A Villiers det 1967, Museum Paris	MNHN
Phalantus feanus	Indonesia, Lesser Sundas 700-800 m alt, N. Lombok lsl PUSUK hill, N slopes of MT. RINJANI, S of BENTEK vill 16-22.3.2009, St Jakl lgt	CAU
Phorastes femoratus	GUYANA: Demerara, Soweyo GDF camp, 50 km SW of Georgetown 27-30 September 1991 J H Martin coll. B M 1991-182	NHM
Pteromalestes nyassae	S. RHODESIA: Odz. diat 11.2.49 N.C.E. Miller. 201	NHM
Rasahus sulcicollis	ECUADOR: Pichincha Prov. Tinalandia Hotel, 10 mi. E of Santa Domingo de los Colovados 16/21 Apr. 1984 leg. Brian Harris MV; LACM ENT 195830	LACM
Thymbreus ocellatus	Teffé. (Ega) Amazones M. de Mathan 2e Trimestre 1879, Distant Coll. 1911-383., NHM 013585808	NHM

Tydides obscurus	BRAZIL, Mato Grosso: 12˚31’N: 55˚37’W Sinop, Oct. 1975, M. Alvarenga	AMNH
Zeraikia novafriburguensis	Rio de Janeiro. Organ Mts. near Tijuca. S. R. Wagner. 1902-287., NHMUK 013586302	NHM
