# Supplementary material for: Revision of the Assassin Bug Genus Sigicoris stat. nov. Based on Morphological Study and Molecular Phylogeny (Heteroptera: Reduviidae: Peiratinae)
Source: Insects. 2022 Oct 19;13(10):951. doi: 10.3390/insects13100951 (PMC9604541; doi:10.3390/insects13100951)
Supplement: Supplementary file 1 [file insects-13-00951-s001.zip › Table S2.pdf]

Table S2. Information of sequences used in phylogenetic study.

Subfamily	Species	Gene	GenBank accession number
Harpactorinae	*Sphedanolestes impressicollis*	COI	KC887536
		16S	KC887536
		Partial 18S	KC413925
Triatominae	*Triatoma rubrofasciata*	COI	MH934953
		16S	MH934953
		Partial 18S	AJ421960
Peiratinae	*Androclus borneensis*	COI*	ON351532
		16S*	ON355293
		Partial 18S*	ON355275
	Androclus granulatus	COI*	ON351533
		16S*	ON355294
		Partial 18S	MH925896
	Brachysandalus lurco	COI*	ON351534
		16S*	ON355295
		Partial 18S*	ON355276
	Calistocoris caesareus	COI*	ON351535
		16S*	ON355296
		Partial 18S*	ON355277
	Catamiarus brevipennis	COI*	ON351536
		16S*	ON355297
		Partial 18S	MH926009
	Ectomocoris luridus	COI*	OP446104
		16S*	OP445248
		Partial 18S*	OP445252
	Ectomocoris quadriguttatus	Partial COI*	OP446105
		16S*	OP445249
		Partial 18S*	OP445253
	Ectomocoris quadrimaculatus	COI*	OP446106
		16S*	OP445250
		Partial 18S*	OP445254
	Ectomocoris xanthopus	COI*	OP446107
		16S*	OP445251
		Partial 18S*	OP445255
	Eidmannia matogrossensis	COI*	ON351539
		16S*	ON355300
		Partial 18S	MH926007
	Fusius dilutus	COI*	ON351540
		16S*	ON355301
		Partial 18S*	ON355279
	Fusius rubricosus	COI*	ON351541
		16S*	ON355302
		Partial 18S	MH925984
	Sigicoris brumalis comb. nov.	COI*	ON351542
		16S*	ON355303
	Sigicoris dominiqueae sp. nov.	COI*	ON351543
		16S*	ON355304

	Partial 18S*	ON355280
Sigicoris sexguttatus comb. nov.	COI*	ON351544
	16S*	ON355305
	Partial 18S*	ON355281
Lamotteus ornatus	COI*	ON351545
	16S*	ON355306
	Partial 18S*	ON355282
Lestomerus affinis	COI*	ON351546
	16S*	ON355307
	Partial 18S	MH925977
Lestomerus sanctus	COI*	ON351547
	16S*	ON355308
	Partial 18S*	ON355283
Melanolestes picipes	COI*	ON351548
	16S*	ON355309
Melanolestes sp.	Partial 18S	KT231841
Microsandalus umbrosus	COI*	ON351549
	16S*	ON355310
	Partial 18S*	ON355284
Neopirates nyassae	COI*	ON351550
	16S*	ON355311
	Partial 18S*	ON355285
Oblongiala zimbabwensis	COI*	ON351551
	16S*	ON355312
	Partial 18S*	ON355286
Pachysandalus collaris	COI*	ON351552
	16S*	ON355313
	Partial 18S*	ON355287
Parapirates cachani	COI*	ON351553
	16S*	ON355314
	Partial 18S*	ON355288
Peirates arcuatus	COI	NC_024264
	16S	NC_024264
Peirates atromaculatus	COI	NC_026670
	16S	NC_026670
Peirates fulvescens	COI	NC_026669
	16S	NC_026669
Peirates lepturoides	COI	NC_026672
	16S	NC_026672
Peirates turpis	COI	NC_026671
	16S	NC_026671
	Partial 18S	KC413804
Phalantus feanus	COI*	ON351554
	16S*	ON355315
	Partial 18S*	ON355289
Phalantus geniculatus	COI	MF806068
	16S	MF806068
Phorastes femoratus	COI*	ON351555
	16S*	ON355316

	Partial 18S*	ON355290
Pteromalestes nyassae	COI*	ON351556
	16S*	ON355317
	Partial 18S	MH926008
Rasahus sulcicollis	COI*	ON351557
	16S*	ON355318
	Partial 18S*	ON355291
Sirthenea flavipes	COI	NC_020143
	16S	NC_020143
	Partial 18S	MH925974
Thymbreus ocellatus	COI*	ON351558
	16S*	ON355319
	Partial 18S*	ON355292
Tydides obscurus	COI*	ON351559
	16S*	ON355320
Zeraikia novafriburguensis	COI*	ON351560
	16S*	ON355321

*: newly-sequenced in present study.
